# Comparative effects of four back squat prescription models on explosive performance and sprint outcomes in resistance-trained men

**DOI:** 10.3389/fphys.2026.1871525

**Published:** 2026-06-11

**Authors:** Junjie Wang, Haoyu Guo, Lingfeng Zhang, Qinfeng Dong

**Affiliations:** 1 School of Physical Education, Ankang University, Ankang, China; 2 School of Basic Medicine Sciences, Capital Medical University, Beijing, China; 3 Department of Physical Education, Sichuan Electronic and Mechanic Vocational College, Panzhihua, China; 4 Department of Public Teaching, Linyi Vocational University of Science and Technology, Linyi, China

**Keywords:** countermovement jump, dynamic strength index, load monitoring, rate of force development, resistance training, sprint performance, training prescription, velocity-based training

## Abstract

**Purpose:**

To compare the effects of maximum intended velocity (MIV), velocity-loss threshold (VLT), target velocity zone combined with velocity-loss threshold (TVZ+VLT), and traditional percentage-based training (PBT) on explosive performance and sprint outcomes in resistance-trained men.

**Methods:**

Fifty-two resistance-trained male athletes were randomly assigned to one of four groups and completed 12 supervised back squat sessions over 4 weeks. Primary outcomes were countermovement jump (CMJ) height, 0–30 m sprint time, and isometric mid-thigh pull rate of force development (IMTP RFD). Secondary outcomes included CMJ peak force and peak power, 0–10 m sprint time, IMTP peak force, relative IMTP peak force, and dynamic strength index (DSI).

**Results:**

Significant baseline-adjusted group effects were retained after Holm correction for CMJ height (F = 7.50, P = .001, ηp² = .324) and 0–30 m sprint time (F = 4.77, P = .011, ηp² = .233). MIV and TVZ+VLT produced greater improvements in CMJ height than PBT, and TVZ+VLT produced a greater improvement in 0–30 m sprint time than PBT. Corrected group effects were also observed for CMJ peak power and DSI, whereas IMTP RFD and IMTP peak force did not show prescription-specific differences.

**Conclusions:**

MIV and TVZ+VLT prescription packages were associated with more favorable short-term changes in selected explosive-performance and sprint-related outcomes than PBT. However, because the interventions differed in velocity zone, relative intensity, movement intent, feedback provision, and velocity-loss termination, and because session-level realized dose data were not retained, these findings should be interpreted as package-level effects rather than evidence for the independent superiority of any isolated VBT component.

## 1 Introduction

Muscular strength and the capacity to express force rapidly are important determinants of many sport actions, including jumping, sprint acceleration, change of direction, and repeated high-intensity mechanical output (Suchomel et al., 2016, 2018). Resistance training is therefore widely used to improve athletic performance, but the adaptive response to a resistance-training program depends not only on exercise selection and weekly frequency, but also on how load, effort, movement velocity, volume, and fatigue are prescribed within each session (Kraemer and Ratamess, 2004; Zourdos et al., 2016). In this context, the method used to regulate back squat training may influence whether adaptations are biased toward maximal force production, explosive power, or sprint-related performance.

Traditional percentage-based training (PBT) remains one of the most common resistance-training prescription models (Sánchez-Medina and González-Badillo, 2011; Weakley et al., 2021). In this approach, external load is typically anchored to a fixed fraction of one-repetition maximum (1RM), and training volume is controlled through planned sets, repetitions, and load progression. PBT is practical and easy to implement, but it assumes that a given percentage of 1RM represents a relatively stable training stimulus across sessions. In practice, daily fluctuations in fatigue, readiness, sleep quality, technical performance, and concurrent training stress may alter the actual stimulus imposed by the same relative load (Behm and Sale, 1993; González-Badillo and Sánchez-Medina, 2010). These limitations have increased interest in velocity-based training (VBT) as a method for monitoring and regulating resistance-training intensity and fatigue.

VBT uses barbell velocity to inform load selection, estimate relative intensity, provide feedback, and monitor within-set fatigue (González-Badillo and Sánchez-Medina, 2010; Sánchez-Medina and González-Badillo, 2011; Weakley et al., 2021). Because movement velocity is closely related to relative load during resistance exercises, and because velocity loss during a set reflects fatigue accumulation, VBT provides a practical framework for autoregulating training in applied sport settings. However, VBT should not be considered a single intervention. Different VBT-derived prescriptions may vary in their emphasis on movement intent, target velocity zone, feedback provision, and velocity-loss termination. Therefore, studies comparing VBT with PBT have provided useful evidence that velocity monitoring can improve load individualization or training outcomes, but they do not fully explain which type of velocity-informed prescription is most appropriate for a given performance goal (Dorrell et al., 2020).

Several VBT-related prescription models are particularly relevant to lower-body strength and power training. Maximum intended velocity (MIV) emphasizes the instruction to move every concentric repetition as fast as possible, even when external load constrains actual barbell velocity; this approach is grounded in the concept that intended movement velocity can influence velocity-specific training adaptations (Behm and Sale, 1993; González-Badillo et al., 2014). Velocity-loss threshold (VLT) training terminates sets once repetition velocity declines beyond a predetermined threshold, thereby regulating fatigue exposure and influencing the balance between mechanical volume, metabolic stress, and velocity quality (Sánchez-Medina and González-Badillo, 2011; Pareja-Blanco et al., 2017; Weakley et al., 2020). Target velocity zone combined with velocity-loss threshold training (TVZ+VLT) uses velocity to guide load selection within a specific velocity range while also limiting within-set velocity loss, which links training to a targeted region of the force-velocity continuum (Samozino et al., 2012; Conceição et al., 2016; Morin and Samozino, 2016). These models differ conceptually and practically from PBT, but they are often discussed under the broad label of VBT. This creates an important research gap, because different velocity-based prescription packages may produce different adaptations in explosive performance, sprint ability, and force-production characteristics.

Recent evidence has increasingly emphasized that velocity- and intent-related variables are not merely monitoring tools, but may shape the adaptive profile of resistance training. Studies in elite or chronically strength-trained athletes have shown that maximal-intended-velocity or ballistic back-squat training can preferentially improve movement velocity, propulsive power, and rapid force-production characteristics, even when maximal strength gains are comparable between training approaches (Lecce et al., 2025a, 2025b). Similarly, work in sprinters suggests that autoregulated VBT may provide advantages over fixed percentage-based loading for selected neuromuscular and sprint-related outcomes (Guo et al., 2026). Collectively, these findings indicate that movement intent, execution velocity, and autoregulated loading are practically relevant prescription factors. However, existing studies have generally compared one velocity-informed approach with a conventional or controlled-velocity condition, rather than directly comparing several applied prescription packages that differ in target velocity zone, velocity-loss termination, feedback, and load-regulation strategy within the same experimental framework. The present study addresses this gap by comparing MIV, VLT, TVZ+VLT, and PBT as integrated back-squat prescription packages, while assessing jump, sprint, and isometric force-production outcomes together.

Therefore, the purpose of this study was to compare the short-term effects of four integrated back-squat prescription packages, namely MIV, VLT, TVZ+VLT, and PBT, on explosive performance, sprint-related outcomes, and isometric force-production characteristics in resistance-trained men. We hypothesized that MIV and TVZ+VLT would produce greater improvements in explosive-performance and sprint-related outcomes than PBT and VLT, whereas maximal isometric force would improve across all conditions.

## 2 Materials and methods

### 2.1 Participants

A repeated-measures analysis-of-variance power calculation was conducted using G*Power. Based on a medium effect size (f = 0.25), α = .05, power = .80, 4 groups, and 2 repeated measurements, a minimum sample size of 48 participants was required. To allow equal group allocation and potential attrition, 52 resistance-trained male athletes were recruited. Participants were resistance-trained male athletes (age: 21.5 ± 1.9 y; relative back squat 1RM: 1.42 ± 0.19 kg·kg−1), with ≥1 year of systematic resistance-training experience and technical proficiency in the free-weight back squat and bench press. Participants were excluded if they reported recent musculoskeletal injury, contraindications to maximal testing or resistance training, use of substances affecting neuromuscular performance, or additional structured training during the intervention. The study was approved by the Institutional Review Board of Ankang University (Approval No.: XIPE-2024-ETH-03-018) and complied with the principles of the Declaration of Helsinki, with written informed consent obtained from all participants.

### 2.2 Experimental design

Our intention was to assess potential differences among 4 back squat prescription models on measures of explosive performance, sprint performance, and isometric force-production characteristics. Fifty-two resistance-trained male athletes were randomly assigned to 4 groups: maximum intended velocity (MIV; n = 13), velocity-loss threshold (VLT; n = 13), target velocity zone combined with velocity-loss threshold (TVZ+VLT; n = 13), and traditional percentage-based training (PBT; n = 13). Group allocation was performed using a computer-generated randomization sequence with equal allocation to the four groups. Baseline characteristics of each group are presented in **Table 1**.

**Table 1:** Participant baseline characteristics by group.

Variable	Unit	MIV (n=13)	VLT (n=13)	TVZ+VLT (n=13)	PBT (n=13)	p
Age	years	21.5 ± 1.9	21.8 ± 2.0	21.2 ± 1.8	21.6 ± 2.1	0.83
Height	cm	176.3 ± 5.2	175.8 ± 6.1	177.1 ± 5.7	176.6 ± 5.9	0.91
Body mass	kg	75.4 ± 7.6	76.3 ± 7.9	74.9 ± 8.2	74.1 ± 7.1	0.88
Body mass index	kg·m^-^²	24.2 ± 1.8	24.1 ± 2.0	24.3 ± 1.9	23.8 ± 1.7	0.94
Training experience	years	2.4 ± 0.9	2.6 ± 1.0	2.3 ± 0.8	2.5 ± 1.1	0.79
Relative back squat 1RM	kg·kg^-^¹	1.43 ± 0.18	1.40 ± 0.20	1.45 ± 0.17	1.41 ± 0.19	0.86

Values are mean ± SD. These baseline characteristics were retained from the original dataset documentation and did not differ significantly between groups.

**Figure 1:**
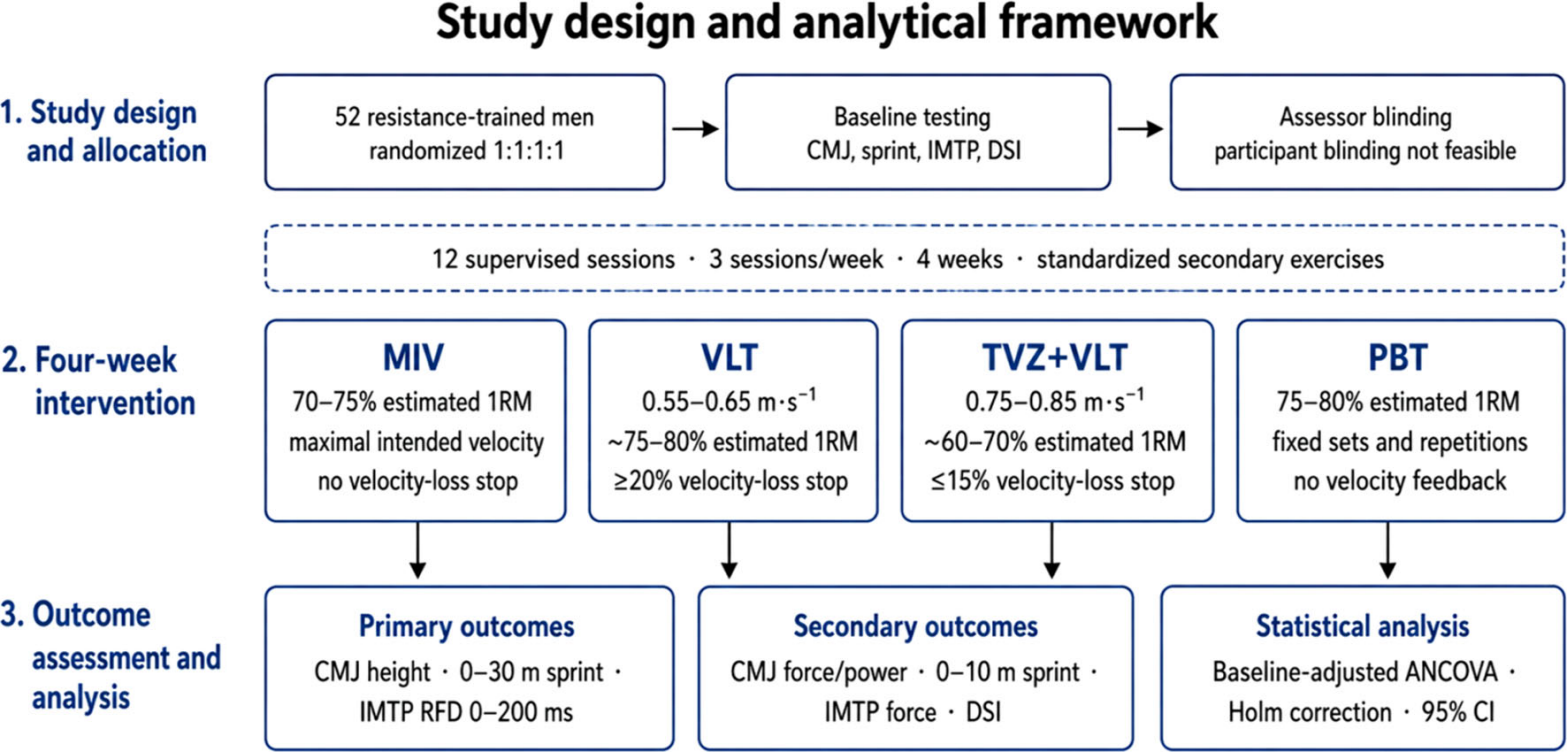
Technical framework of the trial. The image summarizes randomization, four prescription packages, intervention structure, outcome hierarchy, and the statistical interpretation boundary.

Measurements of countermovement jump (CMJ) height, CMJ peak force and peak power, 0–10 m and 0–30 m sprint performance, isometric mid-thigh pull (IMTP) peak force and rate of force development, relative IMTP peak force, dynamic strength index, and load–velocity profiling were taken after familiarization. All participants completed 12 supervised back squat training sessions over a 4-week period, with 3 sessions performed each week and at least 48 hours between sessions. The free-weight back squat was the primary training exercise and was prescribed according to the allocated model. Secondary exercises were standardized across groups and consisted of the Romanian deadlift, leg press, and bench press, performed using the same planned sets, repetitions, and loading scheme. Posttests were conducted using the same procedures and testing order as the pretests. The overall study design is shown in **Figure 1**.

### 2.3 Training intervention

Participants in all groups completed 12 supervised training sessions over a 4-week period, with 3 sessions performed each week and at least 48 hours between sessions. A standardized warm-up was completed before each session. The free-weight back squat was the primary training exercise and was prescribed according to the allocated training model. The planned back squat prescriptions are presented in **Table 2**.

**Table 2:** Planned back squat prescription by group.

Parameter	MIV (n=13)	VLT (n=13)	TVZ+VLT (n=13)	PBT (n=13)
Load basis	% estimated 1RM	Load-velocity profile	Target velocity zone	% estimated 1RM
Intensity weeks 1-2	70-75% 1RM	0.55-0.65 m·s^-^¹ (~75-80% 1RM)	0.75-0.85 m·s^-^¹ (~60-70% 1RM)	75% 1RM
Intensity weeks 3-4	70-75% 1RM	0.55-0.65 m·s^-^¹ (~75-80% 1RM)	0.75-0.85 m·s^-^¹ (~60-70% 1RM)	80% 1RM
Sets	4	4	4	4
Repetitions weeks 1-2	6 fixed	4-8; VLT governed	4-8; VLT governed	6 fixed
Repetitions weeks 3-4	6 fixed	4-8; VLT governed	4-8; VLT governed	5 fixed
Velocity-loss criterion	None	≥20% from reference repetition	≥15% from first repetition	None
Velocity feedback	Yes	Yes	Yes	No

The table describes planned prescription. Actual session-level completed repetitions, tonnage, realized velocity loss, and individual dose variability were not available; therefore, actual training-dose equivalence cannot be verified.

The MIV group performed the back squat with a fixed planned load of approximately 70% to 75% of estimated 1RM. Participants were instructed to complete every concentric repetition with maximal intended velocity, and this instruction was reinforced before and during each set. Sets were not terminated according to a velocity-loss rule. The VLT group used a load selected from a slower target mean concentric velocity range of approximately 0.55 to 0.65 m·s−1. During each set, repetitions were continued until the prescribed velocity-loss threshold was reached. This condition represented a velocity-based prescription model emphasizing fatigue regulation within a heavier, strength-oriented velocity zone. The TVZ+VLT group used a target first-repetition velocity zone of approximately 0.75 to 0.85 m·s−1 combined with velocity-loss set termination. This prescription was designed to regulate both load selection and within-set fatigue while maintaining a higher-velocity training stimulus. The PBT group followed a fixed percentage-based loading model without real-time velocity feedback. Participants completed the prescribed repetitions regardless of barbell velocity. This condition served as the traditional resistance-training comparison group.

Secondary exercises were standardized across groups and consisted of the Romanian deadlift, leg press, and bench press, performed using the same planned sets, repetitions, and loading scheme. Because session-level completed repetitions, total tonnage, mean concentric velocity across all repetitions, and realized velocity loss were not retained, the groups were considered matched at the level of planned training structure and secondary exercise programming rather than verified realized mechanical dose.

### 2.4 Procedures

#### 2.4.1 Countermovement jump

To conduct the CMJ test, participants were instructed to jump as high as possible. The procedure involved starting from a standing position, performing a downward countermovement to a self-selected depth, and then immediately jumping vertically in 1 continuous movement. Participants were instructed to keep their hands on their hips throughout the test to eliminate the influence of arm swing. All jumps were performed on a calibrated force platform, allowing direct measurement of vertical ground reaction forces. CMJ height, propulsion peak force, and peak power were calculated from the force–time record using established impulse–momentum procedures. Each participant completed 3 maximal CMJ trials, with approximately 30 seconds of rest between trials. The trial with the greatest jump height was retained for subsequent analysis (Linthorne, 2001; McMahon et al., 2018).

#### 2.4.2 Sprint testing

Linear sprint performance was assessed over 0–10 m and 0–30 m using electronic timing gates. Before testing, participants completed sprint-specific warm-up efforts at submaximal intensities. Participants started each trial from a standing 2-point stance, with the front foot positioned behind the first timing gate to avoid premature triggering. Each participant completed 2 maximal sprint trials, separated by 3 minutes of passive recovery. Participants were strongly encouraged to sprint maximally through the final timing gate. The fastest 0–10 m and 0–30 m split times were retained for subsequent analysis.

#### 2.4.3 Isometric mid-thigh pull

IMTP testing was used to assess maximal isometric force-production capacity and rate of force development. Participants adopted a standardized mid-thigh pull position with joint angles consistent with established recommendations (Comfort et al., 2018; McMahon et al., 2018). They were instructed to pull “as hard and as fast as possible” against the fixed bar and to sustain maximal effort for approximately 3 seconds. Peak force, relative peak force, and rate of force development over the 0–200 ms epoch from force onset were retained for analysis. Force onset was defined as the point at which vertical force exceeded 5 times the standard deviation of the quiet standing baseline, consistent with established methodological recommendations (Maffiuletti et al., 2016). The best trial, based on the primary force criterion, was used for subsequent analysis.

#### 2.4.4 Load–velocity profiling and estimated 1RM

Participants completed an incremental loading protocol in the free-weight back squat to establish an individual load–velocity profile. Mean concentric velocity was recorded using a linear position transducer. Loads were progressively increased across trials, and participants were instructed to perform the concentric phase with maximal intended velocity while maintaining correct squat technique. The load–velocity relationship was fitted using linear regression, and estimated 1RM was derived from the load corresponding to the minimum velocity threshold for the back squat. The estimated 1RM was then used to guide the training load prescriptions for the relevant groups (González-Badillo and Sánchez-Medina, 2010; Comfort et al., 2015; Sánchez-Medina et al., 2017).

### 2.5 Statistical analysis

The data are presented as mean (SD). Normality was assessed using the Shapiro–Wilk test and visual inspection of quantile–quantile plots, and homogeneity of variance was assessed using Levene’s test. Posttest values were analyzed using analysis of covariance, with group as the fixed factor and the corresponding pretest value as the covariate. Holm correction was applied separately for the primary outcomes, including countermovement jump height, 0–30 m sprint time, and isometric mid-thigh pull rate of force development, and for the secondary outcomes, including countermovement jump peak force and peak power, 0–10 m sprint time, isometric mid-thigh pull peak force, relative isometric mid-thigh pull peak force, and dynamic strength index. Pairwise between-group differences in change scores were expressed as Hedges’ g with 95% confidence intervals. Partial eta-squared was calculated for analysis-of-covariance models, with values of ≥.06 and ≥.14 interpreted as moderate and large effects, respectively. All statistical analyses were performed using IBM SPSS Statistics software, version 29.0. The significance level was set at P <.05 after correction.

## 3 Results

### 3.1 Participant flow and baseline comparability

All 52 randomized participants completed the intervention and were included in the final analysis. Each group contained 13 participants. No adverse events were recorded in the retained study documentation. All participants attended each of the 12 scheduled training sessions; no sessions were missed or required rescheduling. Squat technique was monitored by a qualified strength and conditioning coach throughout each session. No participants reported significant musculoskeletal discomfort or adverse responses attributable to the training intervention. Baseline values did not differ between groups for any analyzed performance outcome, and baseline demographic and training characteristics were similar across groups (**Table 1**). Planned training prescriptions are shown in **Table 2**. Descriptive pre- and post-intervention values and observed changes are summarized by outcome family in **Tables 3** and **4**. Selected pairwise change-score contrasts are reported in **Table 5**.

**Table 3:** Primary outcome change scores and baseline-adjusted group effects.

Outcome	Unit	MIV change [95% CI]	VLT change [95% CI]	TVZ+VLT change [95% CI]	PBT change [95% CI]	F	p	η²
CMJ height	cm	3.79 [2.54, 5.04]	2.10 [1.04, 3.16]	4.21 [3.15, 5.26]	1.40 [0.44, 2.36]	7.50	0.001	0.324
0-30 m sprint	s	-0.14 [-0.20, -0.08]	-0.08 [-0.13, -0.03]	-0.15 [-0.20, -0.10]	-0.05 [-0.11, 0.01]	4.77	0.011	0.233
IMTP RFD 0-200 ms	N·s^-^¹	580.01 [411.38, 748.64]	498.02 [281.60, 714.43]	612.00 [418.15, 805.85]	411.98 [289.29, 534.67]	1.43	0.245	0.084

Sprint-time changes are negative when performance improves. ANCOVA models used post-intervention outcome as the dependent variable, group as fixed factor, and baseline value as covariate. Holm correction was applied within the primary-outcome family.

**Table 4:** Secondary outcome change scores and baseline-adjusted group effects.

Outcome	Unit	MIV change [95% CI]	VLT change [95% CI]	TVZ+VLT change [95% CI]	PBT change [95% CI]	F	p	η²
CMJ peak force	N	196.04 [112.02, 280.05]	125.32 [51.58, 199.07]	185.70 [99.01, 272.39]	96.45 [-7.20, 200.09]	1.75	0.508	0.101
CMJ peak power	W	312.00 [204.65, 419.35]	197.99 [93.72, 302.26]	341.00 [250.00, 432.00]	142.00 [59.89, 224.11]	4.62	0.033	0.228
0-10 m sprint	s	-0.08 [-0.12, -0.04]	-0.05 [-0.10, -0.00]	-0.09 [-0.11, -0.07]	-0.03 [-0.07, 0.01]	3.64	0.077	0.188
IMTP peak force	N	188.98 [108.65, 269.31]	256.00 [145.61, 366.39]	197.98 [94.26, 301.71]	288.99 [171.59, 406.39]	0.94	0.857	0.057
Relative IMTP peak force	N·kg^-^¹	2.49 [1.08, 3.90]	3.38 [1.51, 5.26]	2.61 [0.65, 4.56]	3.89 [2.37, 5.42]	0.85	0.857	0.052
Dynamic Strength Index	AU	0.022 [0.008, 0.035]	-0.020 [-0.037, -0.003]	0.018 [-0.001, 0.038]	-0.038 [-0.058, -0.018]	14.49	<0.001	0.481

Holm correction was applied within the secondary-outcome family. DSI is interpreted as an exploratory derived index rather than as a standalone diagnostic tool.

**Table 5:** Selected pairwise comparisons of change scores.

Outcome	Comparison	Mean difference	95% CI	Hedges g	p
CMJ height	MIV vs PBT	2.39	0.90 to 3.89	1.26	0.012
CMJ height	TVZ+VLT vs PBT	2.81	1.46 to 4.16	1.63	0.001
CMJ height	TVZ+VLT vs VLT	2.11	0.69 to 3.52	1.17	0.016
CMJ height	MIV vs TVZ+VLT	-0.42	-1.96 to 1.13	-0.21	0.585
0-30 m sprint	MIV vs PBT	-0.09	-0.17 to -0.01	-0.90	0.108
0-30 m sprint	TVZ+VLT vs PBT	-0.10	-0.17 to -0.03	-1.11	0.037
0-30 m sprint	TVZ+VLT vs VLT	-0.07	-0.13 to -0.00	-0.84	0.113
0-30 m sprint	MIV vs TVZ+VLT	0.01	-0.06 to 0.08	0.10	0.797
CMJ peak power	MIV vs PBT	170.00	41.97 to 298.03	1.04	0.046
CMJ peak power	TVZ+VLT vs PBT	199.00	82.89 to 315.11	1.34	0.008
CMJ peak power	TVZ+VLT vs VLT	143.01	11.91 to 274.10	0.86	0.101
CMJ peak power	MIV vs TVZ+VLT	-29.00	-162.31 to 104.31	-0.17	0.657
Dynamic Strength Index	MIV vs PBT	0.060	0.037 to 0.083	2.04	<0.001
Dynamic Strength Index	TVZ+VLT vs PBT	0.057	0.030 to 0.084	1.67	<0.001
Dynamic Strength Index	TVZ+VLT vs VLT	0.038	0.014 to 0.063	1.21	0.008
Dynamic Strength Index	MIV vs TVZ+VLT	0.003	-0.020 to 0.026	0.10	0.785
IMTP RFD 0-200 ms	MIV vs PBT	168.02	-29.52 to 365.56	0.67	0.368
IMTP RFD 0-200 ms	TVZ+VLT vs PBT	200.02	-17.30 to 417.33	0.72	0.348
IMTP RFD 0-200 ms	TVZ+VLT vs VLT	113.98	-161.23 to 389.20	0.32	1.000
IMTP RFD 0-200 ms	MIV vs TVZ+VLT	-31.99	-275.37 to 211.38	-0.10	1.000

For sprint time, negative mean differences indicate greater improvement for the first-listed group. Hedges g uses the pooled SD of change scores; interpretation should consider outcome direction.

**Figure 2:**
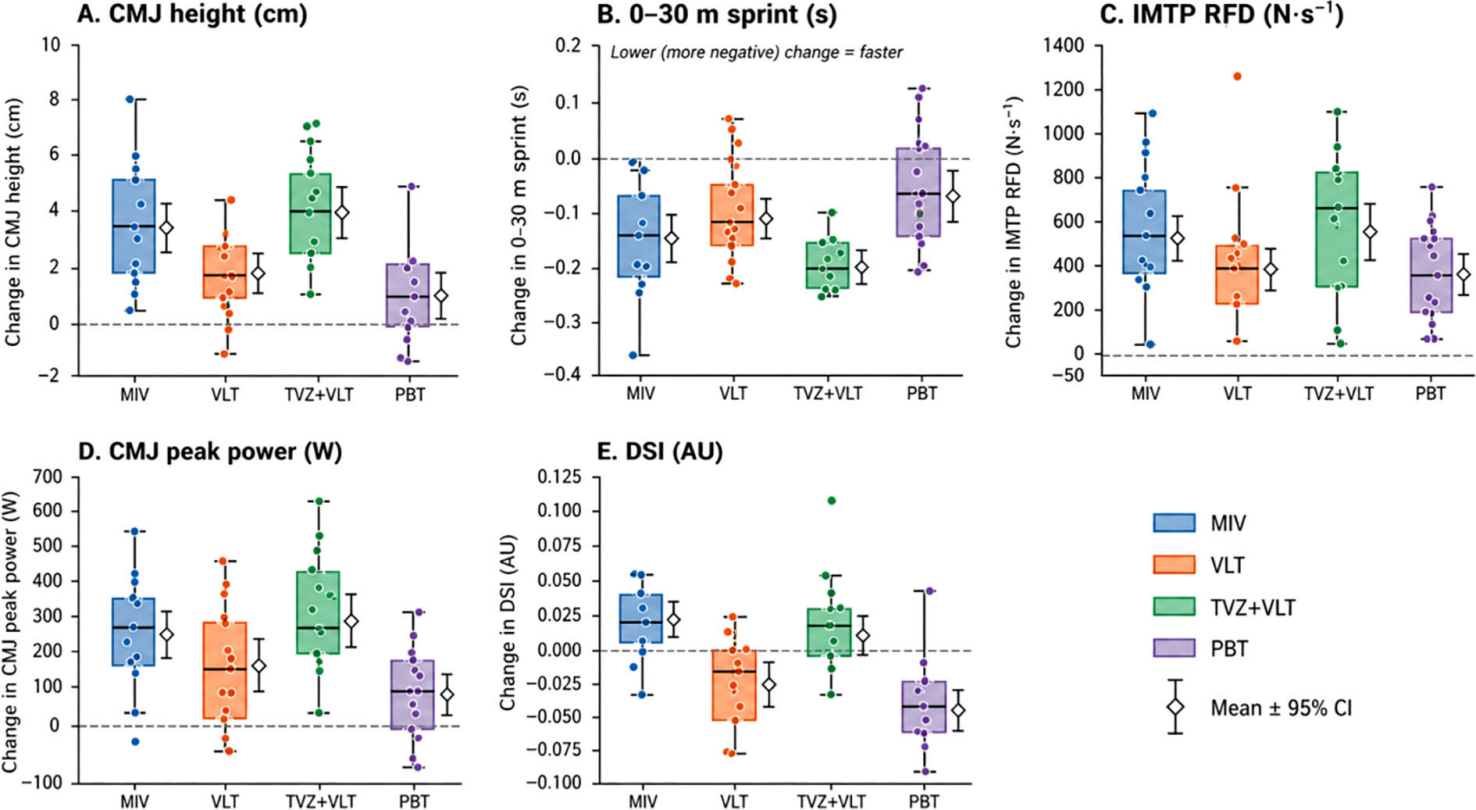
Individual change-score distributions across groups for **(A)** CMJ height, **(B)** 0–30 m sprint time, **(C)** IMTP RFD, **(D)** CMJ peak power, and **(E)** Dynamic Strength Index. Boxplots show median and interquartile range; open circles represent individual participants; diamonds represent group mean with 95% Cl. For 0-30 m sprint time, more negative values indicate faster performance.

**Figure 3:**
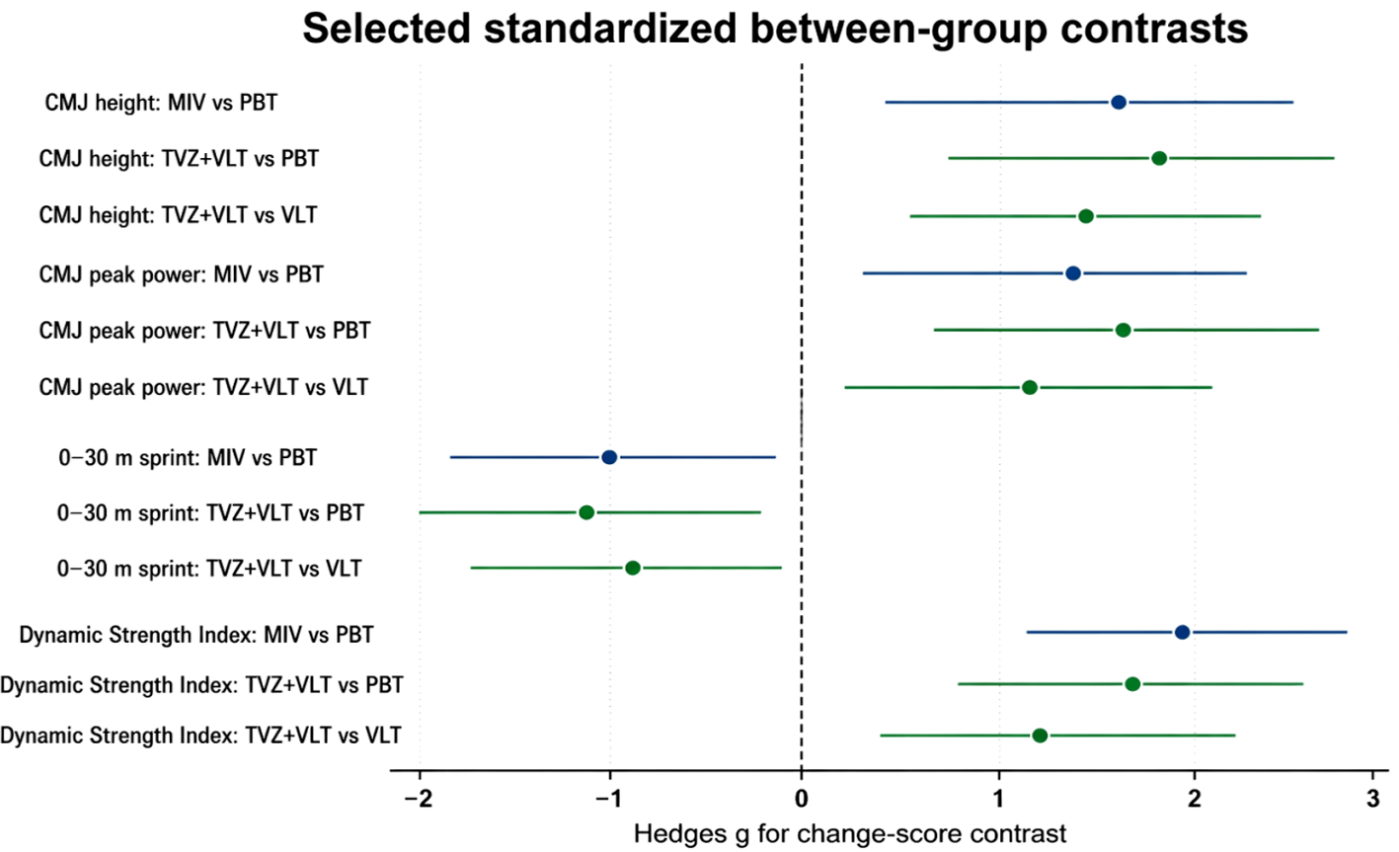
Selected standardized between-group change-score contrasts for key outcomes. Points represent Hedges g and horizontal lines represent approximate 95% confidence intervals derived from the corresponding change-score contrasts. Interpretation should consider the direction of each outcome, especially sprint time.

The results are presented according to the revised hierarchy of outcomes. This is important because several variables changed in the expected direction, but not all retained corrected between-group evidence. The narrative therefore distinguishes between corrected group effects, descriptive within-group improvements, and exploratory patterns. Individual change-score distributions are displayed in **Figure 2**, and standardized pairwise contrasts are displayed in **Figure 3**.

### 3.2 Primary outcomes

For CMJ height, the baseline-adjusted group effect remained significant after Holm correction (F = 7.50, p=0.001, partial ηp²=0.324). Mean changes were +3.79 cm (95% CI: 2.54, 5.04) for MIV, + 2.10 cm (1.04, 3.16) for VLT, + 4.21 cm (3.15, 5.26) for TVZ+VLT, and +1.40 cm (0.44, 2.36) for PBT. Pairwise change-score contrasts indicated greater improvements for MIV versus PBT, TVZ+VLT versus PBT, and TVZ+VLT versus VLT after correction. The MIV versus TVZ+VLT contrast was small and uncertain, and should not be interpreted as evidence that the two prescriptions are equivalent.

For 0–30 m sprint time, the baseline-adjusted group effect also remained significant after Holm correction (F = 4.77, p=0.011, partial ηp²=0.233). Mean changes were -0.139 s (-0.200, -0.078) for MIV, -0.080 s (-0.128, -0.032) for VLT, -0.148 s (-0.196, -0.101) for TVZ+VLT, and -0.050 s (-0.106, 0.006) for PBT. The TVZ+VLT versus PBT pairwise contrast remained after Holm correction. Other pairwise sprint contrasts favored the velocity-emphasized prescriptions numerically, but should be interpreted descriptively because they did not all retain corrected pairwise evidence.

For IMTP RFD, all groups improved from baseline; however, the baseline-adjusted group effect did not remain significant after Holm correction (F = 1.43, p=0.245, partial ηp²=0.084). Accordingly, the manuscript does not claim a reliable between-group advantage for RFD. This result differs from the stronger interpretation that would be obtained from uncorrected or interaction-only analyses and is central to the conservative conclusion of the paper.

### 3.3 Secondary outcomes

Among secondary outcomes, corrected group effects remained for CMJ peak power (F = 4.62, p=0.033, partial ηp²=0.228) and DSI (F = 14.49, p<0.001, partial ηp²=0.481). CMJ peak power increased by +312 W for MIV, + 198 W for VLT, + 341 W for TVZ+VLT, and +142 W for PBT. Pairwise evidence was strongest for TVZ+VLT versus PBT, with other contrasts interpreted as supportive but not definitive.

DSI increased in MIV (+0.022 [0.008, 0.035]) and TVZ+VLT [+0.018 (-0.001, 0.038)] and decreased in VLT [-0.020 (-0.037, -0.003)] and PBT [-0.038 (-0.058, -0.018)]. Pairwise change-score contrasts indicated greater DSI increases in MIV and TVZ+VLT than in VLT and PBT after Holm correction. Because DSI is derived from CMJ and IMTP peak force, this finding is interpreted as exploratory evidence of different force-velocity adaptation profiles rather than as direct evidence for a validated periodization decision rule.

CMJ propulsion peak force, 0–10 m sprint time, IMTP peak force, and relative IMTP peak force did not retain corrected between-group effects. IMTP peak force increased within all groups, with mean changes ranging from approximately +189 N to +289 N, but the adjusted group effect was not significant. Thus, the evidence supports a common improvement in maximal isometric force across training conditions rather than a clear superiority of one prescription model for this outcome over four weeks.

## 4 Discussion

This trial compared four integrated back-squat prescription packages over a four-week intervention in resistance-trained men. The corrected analysis supports a specific and deliberately narrow conclusion: MIV and TVZ+VLT were associated with more favorable short-term changes in selected jump- and sprint-related outcomes than PBT and, for some outcomes, VLT. These findings should not be interpreted as evidence for the independent superiority of movement intent, target velocity-zone selection, feedback provision, or velocity-loss termination alone, because the experimental conditions differed simultaneously across multiple prescription components and session-level realized dose data were not retained. Maximal isometric force improved across all groups without a prescription-specific advantage, and IMTP RFD showed numerically larger changes in MIV and TVZ+VLT but did not retain a corrected between-group effect.

### 4.1 Interpretation as applied training science

The study is best understood as an applied training-science comparison rather than a pure mechanistic physiology experiment. In applied settings, practitioners choose prescription packages that bundle multiple decisions: how load is selected, whether velocity feedback is provided, how effort is cued, when a set is terminated, and how fatigue is constrained. The present study compared such bundled prescriptions, making the design ecologically relevant for strength and conditioning practice.

From a practical perspective, MIV and TVZ+VLT provide more favorable short-term explosive-performance profiles, while all prescriptions improve maximal isometric force. This finding offers coaches a practical decision framework without requiring causal mechanistic specificity: when a mesocycle prioritizes jump and sprint-related qualities, MIV or TVZ+VLT may be more appropriate prescription choices. When maximal force development is the primary goal, heavier velocity-zone VLT or conventional PBT may remain suitable. The key contribution of this study is clarifying which prescription packages are compatible with each training objective over a short block, not establishing a universal hierarchy.

### 4.2 Jump performance and explosive power adaptations

The CMJ height and CMJ peak power findings are consistent with the principle of training specificity. MIV required athletes to attempt maximal concentric velocity on every repetition, whereas TVZ+VLT constrained the working load to a higher velocity region and limited within-set velocity decline. Both approaches plausibly preserved a training stimulus aligned with rapid force expression. Prior work supports the hypothesis that intended velocity can influence velocity-specific adaptation (Behm and Sale, 1993; González-Badillo et al., 2014), and broader power-training literature demonstrates that adaptations are partly specific to the force–velocity characteristics of the exercise stimulus (Cormie et al., 2011; Suchomel et al., 2017). Contemporary evidence further suggests that resistance-training adaptations involve coordinated changes across supraspinal and spinal levels, including spinal motoneurons, neural drive, motor-unit recruitment thresholds, and rate coding, and that movement intent may preferentially influence efferent neural adaptations (Lecce et al., 2026).

The VLT condition also improved CMJ outcomes, though to a lesser extent. This should not be interpreted as evidence that VLT is ineffective. The VLT prescription employed here targeted a slower velocity zone and a higher velocity-loss threshold than TVZ+VLT, likely emphasizing force-oriented loading and fatigue regulation over ballistic velocity quality. This distinction is important: a VLT model can be designed in many ways, and the present VLT condition represents one specific implementation. Future studies should compare different VLT magnitudes within the same velocity zone to separate threshold effects from velocity-zone effects.

### 4.3 Sprint performance

The 0–30 m sprint result suggests that a short block of velocity-emphasized lower-body resistance training may transfer to sprint acceleration. Sprint acceleration requires the capacity to generate large impulses within brief ground-contact times, a capacity related to lower-body strength, power, and rapid force expression (Haugen et al., 2019). The corrected evidence was strongest for TVZ+VLT versus PBT, with MIV also showing a descriptively favorable change. This pattern suggests that constraining training to a higher target velocity zone while limiting velocity loss may provide a stimulus compatible with sprint acceleration requirements. The transfer from back-squat prescription to 0–30 m sprint performance is likely indirect. The back squat primarily develops lower-body force and power under a vertical loading pattern, whereas sprint acceleration depends on the coordinated expression of propulsive impulse, horizontal force orientation, technical execution, and short ground-contact force production (Morin et al., 2011; Contreras et al., 2017). Therefore, the present sprint findings should be interpreted as evidence of transfer from general lower-body force- and power-oriented training rather than as evidence of a direct sprint-technique effect (Seitz et al., 2014).

Sprint adaptation is multi-factorial, and the intervention did not manipulate sprint technique, horizontal force orientation, plyometric exposure, or sport-specific running volume. Sprint improvements should therefore be interpreted as transfer from a lower-body strength training prescription, not as a comprehensive sprint-training effect. The practical magnitude of approximately 0.06–0.10 s over 30 m may be meaningful in competitive contexts, but its interpretation depends on the athlete’s sport, training age, and baseline sprint capacity. The finding supports further study rather than a categorical recommendation.

### 4.4 Rate of force development: the importance of multiplicity correction

The IMTP RFD result is methodologically important. Although all groups improved and MIV and TVZ+VLT showed numerically larger changes than PBT, the baseline-adjusted group effect did not remain significant after Holm correction (*F* = 1.43, *p* = 0.245). Consequently, no reliable between-group RFD advantage can be claimed. This contrasts with the interpretation that would follow from uncorrected interaction-only analyses, and underscores the importance of defining outcome hierarchies and applying appropriate multiplicity control in multi-outcome resistance training studies.

The literature provides plausible biological reasons to expect velocity-emphasized training to influence early-phase RFD, including neural adaptations occurring within short timeframes (Sale, 1988; Folland and Williams, 2007) and the dependence of RFD on early motor unit recruitment and discharge behavior (Aagaard et al., 2002; Maffiuletti et al., 2016; Del Vecchio et al., 2019). However, these mechanisms were not directly measured. The observed numerical pattern is compatible with a neural-adaptation explanation, but the statistical evidence after correction does not support a strong claim. This conservative treatment is scientifically defensible and distinguishes biological plausibility from inferential support in the present dataset.

A contemporary mechanistic framework suggests that resistance-training adaptations involve coordinated changes across supraspinal and spinal regions, spinal motoneurons, and the muscular apparatus. From this perspective, high movement intent and high-velocity execution may preferentially stress neural pathways involved in rapid force expression, including neural drive, motor-unit recruitment thresholds, and discharge behavior. This framework provides a plausible explanation for why MIV and TVZ+VLT showed numerically favorable RFD changes and clearer improvements in jump-related outcomes. However, the present study did not directly measure corticospinal excitability, motor-unit behavior, electromyography, muscle architecture, tendon stiffness, or metabolic responses. Therefore, these mechanisms should be interpreted as biologically plausible explanations rather than direct findings of the current experiment (Lecce et al., 2026).

### 4.5 Maximal isometric force

IMTP peak force increased across all groups without a corrected between-group effect. This is coherent because all participants completed a structured resistance-training intervention involving the back squat and standardized secondary exercises. All conditions included meaningful external loading that would be expected to stimulate maximal force development through neural and task-practice mechanisms. The lack of session-level dose data is particularly relevant here: without complete tonnage and velocity records, it is not possible to determine whether any group accumulated meaningfully more mechanical work. The correct conclusion is that maximal isometric force improved across conditions and did not show a clear prescription-specific difference over four weeks. Longer interventions with complete dose tracking are needed before drawing conclusions about hypertrophy or chronic maximal-strength development.

### 4.6 Dynamic strength index as an exploratory composite outcome

DSI produced one of the clearest corrected group effects, with MIV and TVZ+VLT increasing DSI and VLT and PBT decreasing it. This divergent pattern indicates that the relationship between ballistic and isometric force expression changed differently across conditions, suggesting that velocity-emphasized or intent-emphasized prescriptions may shift the profile toward ballistic force expression, while heavier or fixed loading may increase isometric force without a proportional increase in ballistic peak force.

Nevertheless, DSI should be interpreted carefully. Because it is a ratio, a change in DSI can occur because the numerator changes, the denominator changes, or both. A decrease in DSI is not necessarily negative if it reflects a useful increase in maximal force; similarly, an increase in DSI is not necessarily positive without evidence of sport-performance transfer. The present findings support DSI as an exploratory monitoring variable, not as an independent prescription rule. Coaches should interpret DSI alongside CMJ height, sprint performance, strength measures, technical observations, and the athlete’s current training phase (Suchomel et al., 2020, 2021).

### 4.7 Training-dose interpretation

A central challenge in resistance-training intervention research is that equal prescribed training structure does not guarantee equal realized training dose. The fact that VBT conditions may terminate sets at a certain velocity loss can reduce fatigue exposure but also alter total repetitions and mechanical work. Conversely, fixed PBT conditions preserve planned repetitions but allow unmeasured variation in velocity quality. The present manuscript explicitly separates prescribed dose from realized dose. The planned program can be described precisely; however, realized dose cannot be reconstructed because the dataset lacks completed repetitions per set, total tonnage, set-by-set mean concentric velocity, actual velocity-loss values, and individual variability in fatigue response. This limitation does not invalidate the observed outcome differences, but it prevents the conclusion that all groups received identical internal or mechanical stimuli.

### 4.8 Positioning within the VBT literature

The present findings complement earlier VBT research by demonstrating that the term ‘velocity-based training’ is insufficiently specific when used as a stand-alone intervention label. Prior comparisons of VBT with PBT (Dorrell et al., 2020; Weakley et al., 2021) have asked whether velocity information improves outcomes, but have not resolved how velocity information should be operationalized. A session governed by a velocity-loss termination rule, a session constrained to a target velocity zone, and a session built around maximal intended velocity each impose different movement constraints, feedback demands, and fatigue profiles. The present study contributes most clearly by operationalizing VBT into distinct prescription packages with known structural differences, providing evidence that these packages produce different short-term adaptation profiles.

The observed similarity between MIV and TVZ+VLT on several outcomes should also be interpreted carefully. These prescriptions are not interchangeable in practice. MIV is simple to implement without technology, relying primarily on coaching emphasis on maximal concentric intent. TVZ+VLT requires real-time velocity measurement but offers explicit autoregulation of load selection and fatigue control. Both may arrive at similar short-term performance changes through different practical routes. MIV is best viewed as an intent-dominant strategy; TVZ+VLT as a velocity-quality and autoregulation strategy. This distinction matters for program design even when short-term outcome means do not clearly separate the two approaches. This perspective extends the recent elite-athlete evidence provided by Lecce et al (Lecce et al., 2025a, 2025b)which confirmed that maximal velocity intent and ballistic execution benefit chronically strength-trained individuals, but did not resolve which of several applied prescription structures is most appropriate for a given performance objective. The present findings address this gap at the package level, offering applied evidence that the choice of prescription structure—not simply the presence or absence of velocity monitoring—shapes short-term adaptation profiles.

### 4.9 Limitations

Several limitations constrain interpretation and generalizability of these findings. First, the four-week intervention does not permit conclusions about long-term structural adaptations, hypertrophy, or chronic strength development. Second, the exclusively young male sports-university sample limits generalizability to female athletes, adolescents, older adults, recreationally trained individuals, and elite athletes with substantially higher training ages. Third, session-level realized dose data were not recorded; actual training-dose equivalence cannot be verified, and the prescription packages differed simultaneously in velocity zone, relative intensity, movement intent, and velocity-loss criterion, preventing isolation of individual causal components. Fourth, with nine dependent variables tested, type I error risk for secondary outcomes is elevated even with Holm correction; the outcome hierarchy was applied *post-hoc* to improve interpretive clarity, which is not equivalent to prospective registration. Fifth, participant blinding was impossible, and the presence or absence of velocity feedback may have influenced motivation. Sixth, direct measures of neural drive, motor unit behavior, muscle architecture, tendon stiffness, and metabolic stress were not collected, so mechanistic explanations remain inferential. Seventh, 1RM was estimated from load-velocity profiling rather than directly measured, introducing systematic error. Finally, study-specific reliability data (ICC, CV%) for the testing procedures could not be calculated from the retained dataset; published reliability values support the choice of methods, but reporting study-specific estimates would improve methodological transparency.

### 4.10 Practical applications

When a short resistance-training mesocycle prioritizes improvements in jump height, sprint acceleration, or explosive power, MIV and TVZ+VLT back-squat prescription packages represent practical and evidence-informed choices. MIV requires no velocity-measurement technology and relies on explicit coaching emphasis on maximal concentric intent; it is therefore straightforward to implement in resource-limited settings. TVZ+VLT offers autoregulated load selection and within-set fatigue control, and is best suited to settings where real-time velocity measurement is available. When maximal force development is the primary training objective, heavier velocity-zone VLT or conventional PBT approaches remain appropriate. All recommendations should be interpreted cautiously, as the present findings apply to a 4-week intervention in resistance-trained men and reflect package-level effects rather than the influence of any isolated prescription variable.

## 5 Conclusion

In resistance-trained men, four weeks of MIV and TVZ+VLT back squat prescription were associated with more favorable changes in selected explosive-performance outcomes than PBT and, for some outcomes, VLT. Corrected evidence was retained for CMJ height and 0–30 m sprint time among primary outcomes, and for CMJ peak power and DSI among secondary outcomes. IMTP RFD improved descriptively but did not show a corrected between-group effect, and IMTP peak force improved across all groups without a clear prescription-specific difference. The findings should be interpreted as effects of integrated training packages rather than isolated effects of movement intent, velocity zone, load intensity, or velocity-loss threshold. The absence of session-level realized dose data and prospective registration limits causal and mechanistic inference. Practically, MIV and TVZ+VLT may be considered when a short mesocycle prioritizes jump and sprint-related qualities, whereas heavier VLT or PBT approaches remain relevant when maximal force development is the primary target.

## Data Availability

The raw data supporting the conclusions of this article will be made available by the authors, without undue reservation.
